# Mesenchymal stromal cells derived from exfoliated deciduous teeth express neuronal markers before differentiation induction

**DOI:** 10.1590/1678-7757-2022-0489

**Published:** 2023-04-14

**Authors:** Letícia Fracaro, Agner Henrique Dorigo Hochuli, Ana Helena Selenko, Luiz Guilherme Achcar Capriglione, Paulo Roberto Slud Brofman, Alexandra Cristina Senegaglia

**Affiliations:** 1 Pontificia Universidade Católica do Paraná School of Medicine and Life Sciences - Core for Cell Technology Curitiba PR Brasil Pontificia Universidade Católica do Paraná, School of Medicine and Life Sciences - Core for Cell Technology, Curitiba, PR, Brasil.

**Keywords:** SHED, Regenerative medicine, Dental tissues, Neuronal characterization

## Abstract

**Objective:**

This study aimed to evaluate neuronal markers in stromal cells from human exfoliated deciduous teeth (SHED) and standardize the isolation and characterization of those cells.

**Methodology:**

Healthy primary teeth were collected from children. The cells were isolated by enzymatic digestion with collagenase. By following the International Society for Cell and Gene Therapy (ISCT) guidelines, SHED were characterized by flow cytometry and differentiated into osteogenic, adipogenic, and chondrogenic lineages. Colony-forming unit-fibroblasts (CFU-F) were performed to assess these cells’ potential and efficiency. To clarify the neuronal potential of SHED, the expression of nestin and βIII-tubulin were examined by immunofluorescence and SOX1, SOX2, GFAP, and doublecortin (DCX), nestin, CD56, and CD146 by flow cytometry.

**Results:**

SHED showed mesenchymal stromal cells characteristics, such as adhesion to plastic, positive immunophenotypic profile for CD29, CD44, CD73, CD90, CD105, and CD166 markers, reduced expression for CD14, CD19, CD34, CD45, HLA-DR, and differentiation in three lineages confirmed by staining and gene expression for adipogenic differentiation. The average efficiency of colony formation was 16.69%. SHED expressed the neuronal markers nestin and βIII-tubulin; the fluorescent signal intensity was significantly higher in βIII-tubulin (p<0.0001) compared to nestin. Moreover, SHED expressed DCX, GFAP, nestin, SOX1, SOX2, CD56, CD146, and CD271. Therefore, SHED had a potential for neuronal lineage even without induction with culture medium and specific factors.

**Conclusion:**

SHEDs may be a new therapeutic strategy for regenerating and repairing neuronal cells and tissues.

## Introduction

Mesenchymal stromal cells (MSC) are multipotent adult cells and act in the renovation and maintenance of adult tissues. The main functions of MSC are the self-renewal potential, differentiation capacity in several cell lines, and extensive paracrine and immunomodulatory activity.^1 , 2^ The International Society for Cell and Gene Therapy (ISCT) provides standards for the characterization of MSCs, as follows: adhesion to plastic, fibroblast-like morphology, differentiation into adipogenic, chondrogenic, and osteogenic lineages, positive expression of the markers CD105, CD73, and CD90, and reduced expression of CD45, CD34, CD14, CD11b, CD79 or CD19, and HLA-DR.^2 , 3^ These cells can proliferate *in vitro,* forming round-shaped colonies of fibroblast morphology, leading to colony-forming units-fibroblast (CFU-F).^4^ The colony formation efficiency remains an essential assay for the quality of cells.^5^ The most studied source of MSC is bone marrow. However, bone marrow collection is a painful procedure with safety risks to the health of patients with low income. Thus, other MSC sources have been further investigated, such as the umbilical cord, adipose tissue, and dental tissue, which are easily accessible sources.^1 , 6^

Mammalian teeth are a tissue of ectodermal origin and the MSC present in this tissue can induce neuronal differentiation.^7 , 8^ Stromal cells from human exfoliated deciduous teeth (SHED) in primary teeth that all children loose in the first 6 to 12 years of development have advantages as they are derived from a non-invasive, accessible source, and fall naturally. Moreover, they are considered young/immature, presenting a lower senescence rate of several pathways, a higher proliferation rate, and differentiation capacity in osteogenic lineage compared to dental pulp stromal cells (DPSC) from permanent teeth.^9^

SHED presents a neural crest phenotype and constitutively expresses the markers of neuro-progenitor proteins. Some markers can identify neuronal precursor cells, neurons, and glial cells involved in neurogenesis, such as CD56 (neural cell adhesion molecule), CD146 (melanoma cell adhesion molecule), CD271 (low-affinity nerve growth factor receptor), and intracellular markers such as βIII-tubulin, nestin, GFAP, SOX1, SOX2, and doublecortin (DCX). Different markers indicate the immature cell stage; tracing this neuronal profile could be a strategy to compare the properties and functionalities of SHED with the neural cells.^10^

Storing MSC, especially those derived from dental tissues, in cell banks under good manufacturing practices conditions allows the use of these samples for future benefit in an autologous or even allogeneic application.^11^ Different stromal cell banks specialize in different stromal cell sources, including MSC derived from the bone marrow, umbilical cord, cord blood, adipose tissue, and SHED.^12 , 13^ Collecting and storing SHED can provide advantages compared to cord blood stromal cells: the deciduous teeth is safe to the donor, would usually be discarded, and is more affordable, meaning less than one-third of the cost to store cord blood. As well as MSCs from other sources, SHED can regenerate into solid tissues such as connective, dental, and bone tissue, and demonstrate a remarkable ability to differentiate into neural tissues.^14 , 15^

The possibility of evaluating neuronal markers in SHED before differentiation induction and standardizing the isolation and characterization of those cells would help to develop future strategies for regenerating and repairing neuronal cells/tissues in neurodegenerative diseases.

## Methodology

### Isolation and cultivation of SHED

The study was approved by the local Research Ethics Committee (Approval number: 3.355.573). Healthy primary teeth (n=3) were collected from children at the Dental Clinic of the Pontifícia Universidade Católica of Paraná. The children’s guardians signed the informed consent form authorizing the material’s collection.

A dental surgeon performed the primary teeth collection. Previously, the patient rinsed the mouth with chlorhexidine to remove possible contaminants. The tooth was removed using dental forceps and transferred to a collection tube containing Iscove’s Modified Dulbecco’s Media (IMDM) (Gibco Invitrogen, USA), 1% penicillin/streptomycin (Gibco Invitrogen, USA), 120 ug/ml fluconazole (Isofarma, Brazil), and sodium heparin (Hemofol, Brazil) (5000U/ml). The collected teeth had total root resorption.

To isolate the SHED, the teeth were washed twice in a phosphate-saline buffer (PBS) with 1% penicillin/streptomycin. Then, the pulp was removed with a K file and macerated with a scalpel. The pulp fragments were dissociated with collagenase type II (Invitrogen, USA) under stirring at 37°C for one hour. The cells were cultivated with IMDM and 15% fetal bovine serum (FBS) (Gibco Invitrogen, USA). The cells were stored in an incubator at 37ºC in a humid atmosphere with 5% CO_2_. When the cells reached a confluence from 80% to 90%, enzymatic detachment with 0.25% trypsin/EDTA was performed and re-plated. All the experiments were released in passage four.

### Immunophenotypic characterization

Flow cytometry characterized the membrane markers using commercial antibodies to analyze cell surface markers’ expression. SHED labeling was performed according to Rebelatto, et al.^16^ (2008). The following antibodies were used: CD29-APC (1:20), CD14-FITC (1:20), CD44-FITC (1:20), CD45-FITC (1:20), CD19-FITC (1:20), CD34-APC (1:20), CD105-APC (1:20), CD73-APC (1:33), CD90-PE (1:100), CD166-PerCP (1:33). For viability and apoptosis, 7-AAD and annexin V were used, respectively. IgG1 mouse isotypic antibodies were used as controls (all markers and dyes used are from Becton Dickinson, San Diego, CA, USA). Approximately 100,000 labeled cells were acquired by the FACS Calibur cytometer (Becton Dickinson, USA) with the default parameters and analyzed with FlowJo® software (Flowjo, Ashland, OR, USA, version 10).

### CFU-F assay

In total, 300 MSC were seeded on 6-well culture plates (TPP, Switzerland) in IMDM with 15% FBS. The medium was changed on the fifth day of culture. On the 10^th^ day, the cells were washed with PBS and stained with a 0.3% Crystal violet (Sigma-Aldrich, St. Louis, Missouri, United States) solution for five minutes. The cells were washed with Milli-Q water until the complete removal of excess dye. The number of colonies holding more than 50 cells was counted using a Stereo microscope (Leica Zoom 2000).

### Differentiation

The osteogenic, adipogenic, and chondrogenic differentiation and evaluation were performed according to Fracaro, et al.^17^ (2020). For osteogenic and adipogenic differentiation, SHED were plated (20,000 cells/cm^2^) in a 24-well plate (TPP, Switzerland) and cultured for 21 days with commercial culture media specific to these lineages (hMSC Osteogenic Differentiation Medium Bullet Kit and hMSC Adipogenic Differentiation Medium Bullet Kit - Lonza, USA). The controls were grown with IMDM and 15% FBS.

Due to controversies regarding adipogenic differentiation in dental pulp MSC from permanent teeth, adipose tissue-derived mesenchymal stromal cells were used as a positive control to have more confidence in the results obtained.

For chondrogenic differentiation, micromass culture was performed with 1×10^6^ cells. These cells were cultured with Chondrocyte Differentiation Medium Bullet kit medium (Lonza, USA) supplemented with TGF-β3 (Lonza, USA) for 21 days. The control was grown with IMDM and 15% FBS.

### Stainings

The SHED induced to osteogenic differentiation was fixed to evaluate the calcium crystals, and the *Alizarin Red S* (Sigma-Aldrich, USA) staining was performed. The SHED-induced adipogenic differentiation was fixed to analyze the presence of lipids vacuoles inside the cells. Furthermore, the *Oil Red O* (Sigma-Aldrich, USA) staining was performed. The chondrogenic differentiation was performed by *Toluidine Blue* staining (Sigma-Aldrich, USA). The cells were observed under a bright-field microscope (NIKON Eclipse Ni).

### RT-PCR for the adipogenic genes

The osteogenic and chondrogenic differentiation is more evident than the adipogenic differentiation in SHED. Thus, the reverse transcription-polymerase chain reaction (RT-PCR) was performed to check the adipogenic differentiation, evaluating the expression of the genes CCAAT/enhancer-binding protein alpha (CEBPA), and lipoprotein lipase (LPL). The total RNA was extracted using the PureLink RNA KIT (Invitrogen, USA) following the manufacturer’s instructions. According to the manufacturer’s instructions, reverse transcription was performed using the High-Capacity cDNA Reverse Transcription Kit with RNase Inhibitor Kit (Applied Biosystems, USA). Polymerase chain reaction (PCR) was carried out with 1ng of cDNA template, 1µM of each primer, Taq polymerase, and reaction mix (Promega, USA).

The following primers were used: QuantiTect Primer Assays Hs_CEBPA_1_SG and Hs_LPL_1_SG from Qiagen (USA). Moreover, 15μL of the RT-PCR products were subjected to electrophoresis in a 2% agarose gel stained with UniSafe Dye Nucleotide Acid Staining Solution (Uniscience, USA) and photographed under ultraviolet transillumination (Axygen Gel Documentation System, USA). Glyceraldehyde-3-phosphate dehydrogenase (GAPDH) transcript was used as an internal control (5′CGTCTTCACCACCATGGAGA3′ and 5′CGGCCATCACGCCACAGTTT3′).

### Evaluation of neuronal marker expression / Immunofluorescence

Immunofluorescence was performed to confirm the expression of the neuronal proteins nestin and βIII-tubulin in SHED. After cell culture, the cells were fixed and washed with a 10 mM Tris Buffered Saline solution containing 0.1% Triton X-100. For blocking solution, 10 mM TBS containing 1% Triton X-100 and 5% goat serum were used for one hour. Then, the samples were incubated with the primary anti-nestin (1:200) (ThermoFisher, USA) and anti-βIII-tubulin (1:800) (Santa Cruz) antibodies at room temperature for one hour. The samples were washed and incubated with a secondary anti-rabbit TRITC (1:350) (Sigma-Aldrich, USA) for nestin and anti-mouse FITC (1:300) (Sigma-Aldrich, USA) for βIII-tubulin at room temperature for one hour. The samples were washed, and the nuclei were stained with 2-(4-aminophenyl)-1H-indole-6-carboxamidine (DAPI) (Sigma-Aldrich, USA).

The fluorescence intensity was quantified to compare and evaluate a difference in the expression of the βIII-tubulin and nestin. As Jensen^18^ (2013) described, fluorescence intensity quantification was performed using ImageJ software developed by Wayne Rasband, version 2.0 for Windows, Cambridge – UK. Five images from each sample were selected and configured in 8 bits (Image-Type-8-bit). To quantify the intensity of the fluorescent signal, the histogram (Image – Adjust – Threshold) was used with the following selected settings: “Default” and “Dark Background.” Results were normalized by dividing the fluorescence intensity by the number of nuclei (DAPI staining) in each image analyzed. Data were expressed as percentage. The fluorescence quantification data were expressed as mean and standard deviation.

### Evaluation of neuronal marker expression / Flow Cytometry

The immunophenotype characterization with specific neuronal markers was performed by flow cytometry. Samples were processed as described in the commercial BD Stemflow Human Neural Lineage Analysis kit (BD Bioscience, USA). Cells were dissociated with StemPro^®^ Accutase^®^ (Gibco, USA) for four minutes, washed with PBS (Gibco, USA), and fixed with BD Cytofix^TM^ (BD Bioscience, USA) for 20 minutes at room temperature. The samples were washed with PBS (Gibco^TM^, USA) and permeabilized with BD Phosflow^TM^ Perm Buffer III (BD Bioscience, USA) for 30 minutes on ice. Cells were washed with wash buffer and incubated in the dark for 30 minutes with the following antibodies: SOX1, SOX2, GFAP, DCX, and nestin. Moreover, the cells were labeled for CD56, CD146, and CD271 markers. Mouse IgG1 isotypic antibodies were used as a control. The cells were acquired by the FACS Calibur flow cytometer (BD Bioscience, USA) and analyzed using the FlowJo^®^ software.

### Statistical analyses

The data were processed, and statistical significance was estimated using GraphPad Prism software version 9.0.0 for macOS (GraphPad, La Jolla, CA, USA). Data are expressed as mean (±), median, average, standard error (SEM), or standard deviation (SD). For all experiments, the D’Agostino & Pearson normality test was performed, and statistical significance was figured out using the student’s t-test of equal variance or the Mann Whitney U test; p values: n.s=p>0.05; *=p<0.05; **=p<0.01; ***=p<0.005; ****=p<0.0001.

## Results

### SHED showed MSC characteristics

**Figure 1:**
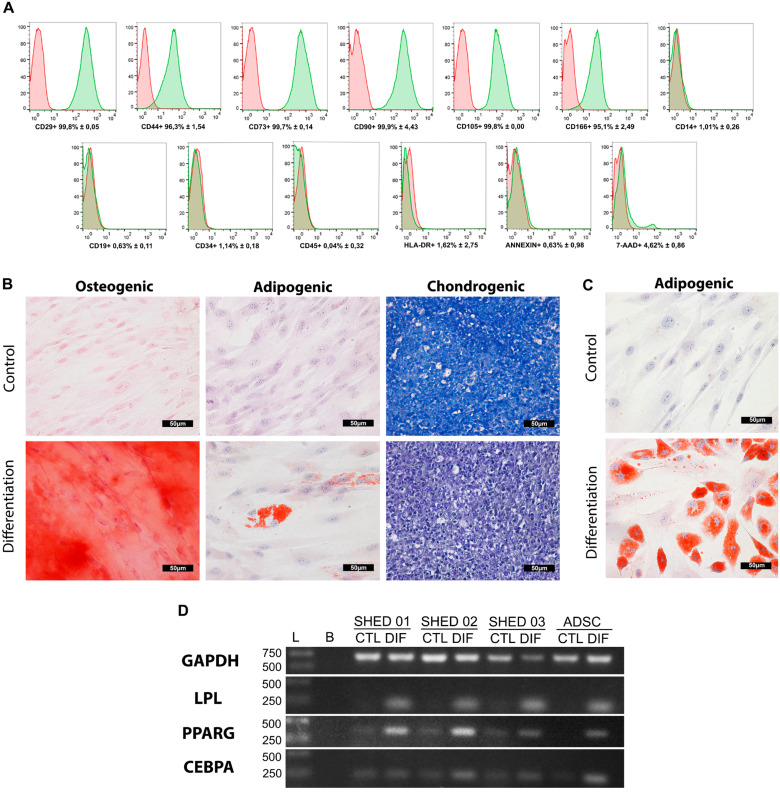
Characterization of SHED (A) Immunophenotypic analysis by flow cytometry representative of the SHED. The green histograms indicate the percentage of the positive population for each antibody. The red histograms indicate the isotypic control of the antibodies. For the analysis of 7-AAD, autologous control was used. Results regarding SHED samples’ immunophenotypic characterization (mean and standard deviation). (B) In vitro differentiation of SHED. Representative image. Osteogenic lineage: control; differentiation; presence of calcium crystals in red. Staining: Alizarin Red S. Adipogenic lineage: control; differentiation; presence of lipid droplets in red. Staining: Oil Red O. Chondrogenic lineage: control; differentiation; cuboidal cells (arrows); and gaps around young chondrocytes. Staining: toluidine blue. Magnification: 400×. Scalebar: 50μm. (C) Representative image of mesenchymal stromal cells derived from adipose tissue used as a positive control for adipogenic differentiation; control; differentiation; the presence of lipids droplets in red. Staining: Oil Red O. (D) Expression of LPL, PPARG and CEBPA in SHED before and after adipogenic induction differentiation by RT-PCR in 2% agarose gel. The GAPDH was used as a constitutive housekeeping gene to normalize changes in specific gene expression. Mesenchymal stromal cells derived from adipose tissue (ADSC) were used as a positive control

SHED showed fibroblast morphology and adhesion to plastic. Flow cytometric analysis of markers related to MSC revealed that cells positively expressed the CD29, CD44, CD73, CD90, CD105, and CD166 markers (>90%), and negatively the CD14, CD19, CD34, CD45, HLA-DR (<4%) ( Figure 1A ).

We observed osteogenic differentiation, as SHED presented calcium crystals stained red after 21 days ( Figure 1B ). The chondrogenic differentiation was evaluated by Toluidine Blue staining. The cells induced to chondrogenic differentiation changed their morphology to a cuboidal shape. Furthermore, we observed the presence of proteoglycans and gaps around the young chondrocytes ( Figure 1B ). In the negative control samples, we found none of these characteristics ( Figure 1B ). The osteogenic and chondrogenic differentiation potential in SHED is evident. On the other hand, the adipogenic differentiation staining by Oil Red O was unexpressive ( Figure 1B ). However, the gene expression analyses show that the SHED expressed different adipogenic genes before and after the adipogenic differentiation ( Figure 1D ).

### SHED had the potential to form colonies

**Figure 2:**
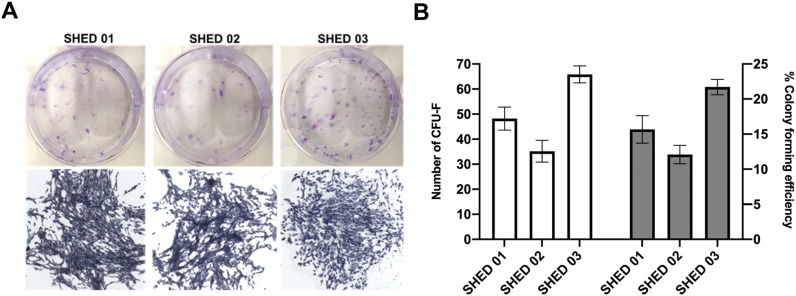
Fibroblast colony-forming units (CFU-F) (A) Representative images of SHED colonies staining by Crystal Violet. Magnification: 40×. (B). The graph shows the number of CFU-F of each sample (white columns) and the colony-forming efficiency (grey columns) on each sample after 10 days of culture. Data are expressed in mean and standard deviation

After cultivating SHED for 10 days, the cells formed colonies ( Figure 2A ). The average number of colonies of the samples was 48±4.59 (SHED 01), 35±4.34 (SHED 02), and 65±3.40 (SHED 03) ( Figure 2B ). The colony formation efficiency of the samples varied between 12.09% (SHED 02) and 21.71% (SHED 03) ( Figure 2B ).

### Neuronal markers are expressed in the SHED without neuronal induction

**Figure 3:**
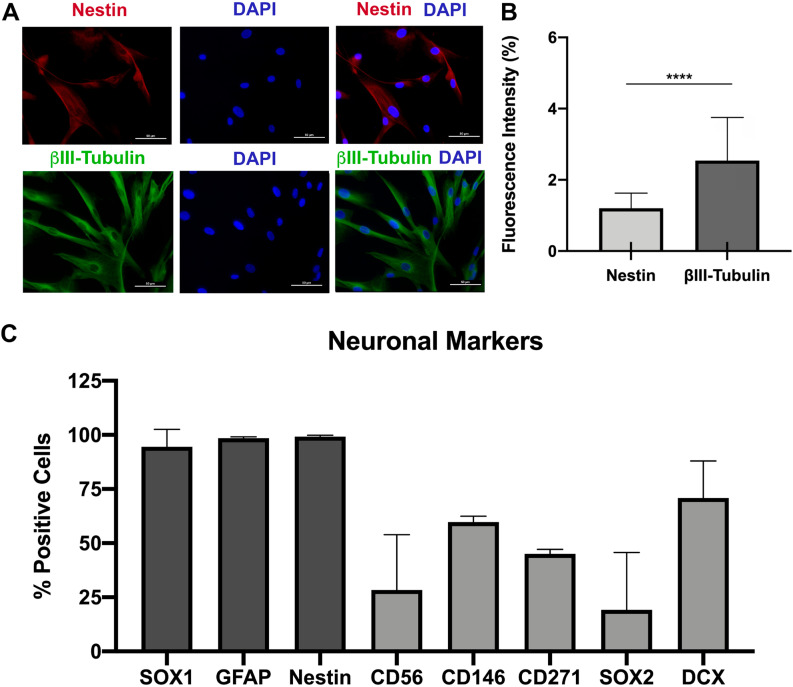
Expression of neuronal markers in SHED (A) Immunofluorescence representative images of SHED labeled with anti-nestin (red marker) and with anti-βIII-tubulin (green marker). The nucleus is blue (DAPI). (B) Quantification of the fluorescence intensity of nestin and βIII-tubulin. (C) Flow cytometry analyses of the positive SHED to neuronal markers (SOX1, GFAP, Nestin, CD56, CD146, CD271, SOX2, and Doublecortin (DCX)). Data expressed as mean and standard deviation ****p<0.0001. 400× magnification. Scale bar: 50µm

To clarify the neuronal potential of SHED, we examined the expression of nestin and βIII-tubulin. The intensity of both markers was measured and quantified. Regarding neuronal markers, the βIII-tubulin, which is specific neuron tubulin, we observed a higher expression when comparing nestin with a significant difference (p<0.0001) ( Figure 3A and Figure 3B ). SHED also had a positive expression (>90%) of SOX1, GFAP, nestin, and expression of CD56, CD146, CD271, SOX2, and DCX (<90%) ( Figure 3C ). Therefore, SHED had a potential for the neuronal lineage even without induction with culture medium and specific factors.

## Discussion

SHED isolation efficiency was 100% and we observed no contamination during cell cultivation. Alansary, et al.^19^ (2020) collected teeth with different levels of root resorption and observed contamination in the culture of half of the samples, even using antibiotics and antimycotics. The authors claimed that teeth with advanced root resorption have a higher chance of contamination during cultivation. However, we did not observe this in our study, because we used teeth with total root resorption, demonstrating that the level of root resorption does not interfere with sample contamination. Since it is not related to root resorption, the technique or the extensive manipulation of the samples may have resulted in the contamination in the studies mentioned.

SHED showed fibroblast-like morphology, plastic adherence, positive immunophenotypic profile for CD73, CD90, CD105, and reduced expression of CD14, CD19, CD34, CD45, and HLA-DR, according to the minimum criteria set up by ISCT to define a population of MSC.^2^ Studies observed a lower expression of CD105 in SHED, an important marker of the panel suggested by the ISCT.^2 , 19 – 21^ This low expression is possibly due to the time of teeth collection (stages of root resorption) and the different isolation and culture methods. According to Alansany, et al.^19^ (2020), the lowest expression of CD105 occurs in cells grown in serum-free conditions. However, based on so many variables, more studies on CD105 must be carried out to understand better which variable acts in the expression. Other studies that used stromal cells from deciduous tooth pulp also found the positive expression of the MSC markers CD29, CD44, and CD166, confirming the similarity to MSC derived from other tissues.^22 , 23^ In this study, all negative markers showed low expression setting up by Dominici, et al.^2^ (2006). On the other hand, the other studies cited did not follow the ISCT guidelines (less than 2%). Alansary, et al.^19^ (2020) used a cocktail of negative markers, and could not identify which marker had an expression above 2%. Rossano, et al.^20^ (2017) showed a higher expression in CD34.

The capacity of the SHED to deposit crystals of calcium confirms the ability of SHED to differentiate in osteogenic lineage, which was evaluated by several techniques.^24 , 25^ We could also evaluate the chondrogenic potential of SHED, showing cuboidal morphology, proteoglycans, and gaps around young chondrocytes, as previously demonstrated by other studies.^26 , 27^

The adipogenic potential of MSC derived from ectoderm origin has some controversies, encouraging us to find how SHED responds to adipogenic stimulus. Some authors only evaluate the adipogenic capacity by observing the staining assays. However, it is impossible to visualize the lipids droplets in all stained cells. Adipogenic gene expression has been reported before and after adipogenic induction in SHED.^8 , 28^ In our study, the gene data revealed that the LPL, expressed in preadipocytes and essential in lipid metabolism and concentration of triglycerides,^29^ is also expressed in SHED induced to adipogenic differentiation. Thus, the cells respond in some way to adipogenic differentiation-inducing factors, suggesting that SHED are a preadipocyte. We observed the same pattern in our positive control.

On the other hand, the CEBPA gene, expressed in the mature adipocytes, seems to be upregulated in SHED 02 induced adipogenic differentiation and the positive control (ADSC) compared to the other samples of SHED. The genotype does not correlate to the phenotype after the adipogenic differentiation in SHED because these cells showed heterogenous lipid droplets. Regarding the results obtained in this study, SHED does not express homogeneous lipid droplets. Therefore, we strongly recommend performing gene expression analysis to assess the adipogenic differentiation, since there may be a difference between the phenotype and the genotype.

The CFU-F confirms that clonogenicity can generate identical stromal cells with the appropriate cell morphology, a consistent feature of MSCs.^30^ This study observed a difference between the average number of colonies and colony formation efficiency, possibly related to the samples’ variability. Several authors carried out the SHED colony-forming units-fibroblast. However, the cell plating density differs between studies, making it difficult to compare results. Despite the difficulty of comparison, all studies showed the potential to form colonies and functionality of SHED.^31 – 33^

The CD56 and CD146 indicate a potential for neuronal differentiation and are expressed in cells that migrate from the neural crest. They are significantly expressed in the central nervous system, contributing to several neuronal functions, migration, proliferation, survival, and differentiation.^34^ The CD56 expression was already evaluated in DPSC, and the cells had a low expression, probably because the enzymes used were different from this study, isolating another type of cells.^34^ The CD56 is a neural crest marker. Consequently, the expression of CD56 in SHED indicates a great potential to induce these cells to neuronal differentiation, since dental pulp shares a common origin, the neural crest, with neuronal cells.

The CD146 membrane marker is a cell adhesion molecule and its expression is related to the activation of intracellular signaling pathways in the brain.^35^ The expression of CD146 in SHED could be related to the neural crest cell migration during embryonic development. Ma, et al.^33^ (2021) suggest that CD146 may be related to the quality of SHED. According to these authors, the higher expression of CD146 is related to a higher rate of proliferation, osteogenic differentiation, and immunoregulatory potential *in vivo* . Thus, the expression of CD146 is related to the potency of dental pulp MSC, both permanent and deciduous. Ma, et al.^33^ (2021) observed that when SHED has more than 30% expression of CD146, these cells have a better result in immunomodulation *in vivo* .

The expression of CD271 (receptor for neurotrophins, which stimulate neuronal cells to survive and differentiate) was already observed in SHED by other authors.^36^ Some studies describe the low expression of CD271 in MSC from other dental tissues.^37^ Moreover, cells that express CD271 have a better ability to agglomerate in neurospheres and differentiate into astrocytes, neurons, and oligodendrocytes.^8^

SHED expressed nestin and βIII-tubulin, markers common to neuronal precursors and immature neurons. This expression may be due to its ectodermal origin, the exact origin of neuronal cells. These markers are absent in the undifferentiated state of sources of mesodermal origin, such as adipose tissue and umbilical cord tissue.^38^ Nestin is a protein in the cytoskeleton, classified as an intermediate filament, initially described in neural stromal cells, cells in development, and adult brains.^39^ Alansary, et al.^19^ (2020) showed a percentage of nestin expression in SHED close to what was observed in our study. Other authors confirmed that cells that express nestin could be considered neural progenitors.^40^ βIII-tubulin is a marker of neuronal cells in the developing and mature human nervous system.^41^ The location of this protein in the cytoskeleton confirms neurons’ identity.^42^ Even without neuronal differentiation, the nestin and βIII-tubulin expression in SHED confirm these cells’ neuronal origins and the cytoskeleton proteins like neuronal cells.

Other markers were used to trace the profile of neural precursor cells, such as SOX1, SOX2, GFAP, and DCX. This study shows the expression of these neuronal markers in SHED. The expression of SOX1 in SHED was never described. SHED expressed this marker because SOX1 acts mainly in neurogenesis and is expressed in neural precursor cells. The expression of the SOX2 marker, also known as the pluripotency marker, has already been reported in SHED.^19 , 28^

SHED expressed astrocytes (GFAP) and neuronal intracellular microtubule proteins such as DCX, which may be related to the SHED neural crest’s origin, giving a greater differentiation ability in neuronal cells.^43^ The DCX expression gives SHED a good advantage compared to bone marrow MSC, which has the mesodermal germ, which does not express DCX.^44^

## Conclusion

Understanding and elucidating the potential of SHED to express neuronal markers before inducing neuronal differentiation is essential to develop simpler treatment protocols and to transfer them from bench to bedside. Among the advantages of using this source of mesenchymal stromal cells is the accessible collection, which is a non-invasive source with ethical criteria that follows the criteria suggested by ISCT.

The storage of different MSCs sources in cell banks is suggested since each source has a different potential. The MSCs are essential in advancing regenerative medicine to treat diseases. Therefore, stromal cell banks can accelerate the safe and efficient use of these cells. SHEDs have enormous potential since they have the same embryonic origin as neurons and express neuronal markers even in the native stage. These characteristics make them an important source for future use in treating neurodegenerative diseases.
